# Money plant disease atlas: A comprehensive dataset for disease classification in ornamental horticulture

**DOI:** 10.1016/j.dib.2024.111216

**Published:** 2024-12-10

**Authors:** Md. Seyam ali Biswas, MD Hasan Ahmad, Sajib Bormon, Rimon, Sohanur Rahman Sohag, Amatul Bushra Akhi

**Affiliations:** Department of Computer Science and Engineering, Daffodil International University, Dhaka, Bangladesh

**Keywords:** Money leaf dataset, Image pre-processing, Image identification, Ornamental agriculture, Transformer, Deep learning

## Abstract

Epipremnum aureum, sometimes known as the Money Plant, is a popular houseplant known for its hearts-shaped leaves and durability. Commonly referred to as Golden Pothos or Devil's Ivy, it is also appreciated for its ornamental value and air cleaning ability. They say that these plants are attractive to many people owing to their tolerance to several conditions and easy care, therefore, it is no surprise that they are found in many households and workplaces. Money Plants are hardy, but like any other plant they can also be infected by various diseases, which may render them less attractive, or even unattractive. This work encompasses bacterial wilt, manganese poisoning aspects and together with a healthy leaves aspect presents all prevalent masses and offer a comprehensive image of diseases. A dataset of 224 × 224 pixel images is utilized to accomplish this work with the intention to further enhance support in Ornamental Horticulture practices and diagnose more accurately. This work not only contributes ideas and approaches in understanding the field of plants pathology but also stresses on the fact how image processing can be beneficial in looking after plants. The dataset serves as a solid foundation for deep learning approaches into Ornamental Agriculture and provides useful insights for researchers studying the cultivation of money plants.

Specifications Table

**Table:** 

Subject	Computer Science
Specific subject area	Image Pre-processing, Image Detection, Image Categorization, Disease classification*.*
Type of data	Image.
Data collection	A collection of good quality images of Money Plants was gathered from various sites, offices and homes, Dhaka, Bangladesh between September 2023 and December 2023. This effort was undertaken in collaboration with an expert from the Ministry of Agriculture, Bangladesh
Data source location	**Location:** Bangladesh Agricultural Development Corporation. **The area:** Kashimpur, Gazipur. **Country:** Bangladesh
Data accessibility	Repository name: Mendeley Data Data identification number: 10.17632/rzjww3vdxt.3 Direct URL to data: https://data.mendeley.com/datasets/rzjww3vdxt/3
Related research article	**None**

## 1 Value of the Data

Epipremnum aureum, popularly called Devil's ivy, Golden images, or Money Plant, is a common houseplant that is appreciated for its durability as well as its heart shaped leaves [1]. Known not only for its pleasing aesthetic but also for its air purifying qualities, the Money Plant has become common in almost every household and business place. Gardeners grow it mainly due to its low care requirements and ability to grow in many environmental conditions. However, contrary to their remarkable hardiness, the Money Plant is prone to a variety of pathogens, ornamental horticulture which affect its appearance and general health and physical well-being [2,3].

Recent developments in machine learning and image processing have created new opportunities for the early diagnosis of Money Plant diseases. This work uses these technologies to create a dataset aiming at precisely spotting and diagnosis of leaf illnesses in the Money Plant [4]. This study provides a thorough review of the different maladies that afflict the Money Plant by focusing on typical difficulties including bacterial wilt and manganese poisoning, as well as a category for healthy leaves.

The dataset, which was collected, assures such level of homogeneity and consistency as it is accompanied by careful data preprocessing that includes the processes of image segmentation, noise removal, scaling and normalisation. Additionally, we have used the methods of data augmentation to balance classes and expand the dataset favorably for the strong development of deep learning systems.

The purpose of this work is to supporting early disease identification and treatment in the Money Plant, the suggested approach emphasises the possibilities of modern technologies in horticultural upkeep. This work emphasizes the significant role of deep learning in modern ornamental horticulture and provides a valuable resource for scholars and practitioners, thereby contributing to the broader field of decorative plant cultivation focusing on the money plant dataset.

As we couldn't find any existing accessible money plant dataset, hence we compared this work with plant leaf disease datasets, and the recently gathered dataset has a number of advantages. This dataset's very clean, shadow-free, noise-free images improve the accuracy of disease detection algorithms by themselves. More exact segmentation and feature extraction made possible by the high quality of the images enable the development of successful deep learning models depend on these processes. This guarantees that academics and practitioners may depend on the dataset for consistent and repeatable results, therefore improving the subject of plant pathology and helping to improve agricultural methods.

## 2 Background

The collection of this dataset resulted from the necessity to solve difficulties in recognising developmental phases of money plant disease common in ornamental agriculture. The development of the dataset fits very nicely with ongoing research in the field of precision cultivation, which employ technical interventions to enhance agricultural practices [5]. This function also motivated us since it was challenging to create accurate detection models without thorough datasets particular to money plant diseases. We have collected 1872 images showing various states; this dataset is an important tool for deep learning algorithm validation and training, and it facilitates quick and precise identification of diseases affecting money plants. By allowing researchers as well as practitioners to access raw data, the dataset article improves a related research publication so increasing reproducibility, transparency, and the possibility of further study to optimise agricultural practices.

## 3 Data Description

The images in the dataset include both disease and newly fallen leaves, representing various stages of the money plant development process. Under the supervision of a subject matter expert, these images were manually taken between September 2023 and December 2023 from the Bangladesh Agricultural Development Corporation's demonstration garden at Kashimpur, Gazipur using the cameras of a Redmi Note 11 Pro Plus & a Samsung S22 smartphone. We have considered only one garden, multiple office, homes for the collection of this dataset in the same area (Kashimpur, latitude and longitude: 23.9778° N, 90.3225° E). The final images were captured and kept in JPG format, with 424 × 424 pixels in size. The dataset's images are labelled with the diseases that correspond with them, making classification and analysis simple:

1. Taking images in noisy environments and with uneven lighting was our main source of difficulty when gathering data.
2. The overlapping leaves and thick vegetation of the money plant made it challenging to get clear and distinct images of particular leaves.
3. To maintain consistency in the dataset, careful positioning and framing were necessary due to the variation in leaf size and shape.
4. The glossy leaves of the money plant produced glare and reflections, which made taking images more difficult
5. The images' quality and usability suffered from occasional pest presence on the leaves impacted.

In this study we have presented three types of money plant leaves. We have taken the help of an agriculture officer to accurately label the dataset, ensuring expert validation of disease categories. We have also applied preprocessing techniques along with data augmentation to address class imbalances. This approach has enhanced model robustness and mitigated the effects of oversampling. The classes include fresh leaves, manganese, and bacterial wilt. Manganese deficiency and bacterial wilt were specifically chosen for this research because of their prevalent and distinct impacts on ornamental plants, particularly the money plant, making them key diseases to focus on for early detection and management. These diseases present unique visual and physiological symptoms that make them suitable for classification using image datasets. Table 1 shows the particulars of these several types of money plant leaves.

**Table 1:** Information regarding the dataset's money plant disease types.

Variety Name	Description	Visualization
Bacterial wilt	Bacterial wilt disease in money plants is characterized by brown, decaying leaf edges that progressively affect the entire leaf. The disease results in the vascular system of the plant getting infected by bacteria that are carried by the soil, which causes the leaves to wilt and turn brown. The infected leaves exhibit a distinctive wilted appearance, which eventually results in the plant's decline if not managed properly [5]	
Manganese Toxicity	Money plants that exhibit interveinal chlorosis, a condition in which the veins remain green but the areas between the leaves turn yellow, are toxic to manganese. This condition is often due to excessive manganese in the soil, leading to nutrient imbalances. Affected leaves may also show crinkling or curling as the toxicity levels increase [6].	
Healthy	Healthy money plant leaves are characterized by their vibrant green color and glossy surface. These leaves are free from any visible diseases, spots, or discolorations. A healthy leaf signifies optimal growing conditions and proper care, reflecting the plant's overall vigor and health [7].	

Automation is a revolutionary tool in the field of agricultural science that could help the ornamental agriculture economy in farming. One of the key benefits is the increase of quality. Automation in jobs like identify the type of disease in the money plant makes a uniform and highly competent final outcome possible. Matching consumer demands and global markets expectations is imperative, as they frequently have elevated quality standards. Although personally identifying the disease is still rather common, it is well known to have several drawbacks. Human perspective is prone to mistakes and conflicts since it is subjective and affected by factors like tiredness and personal judgment. It additionally requires a lot of time, especially when managing more leaves, which might cause inefficiencies and higher labor expenses. These systems identify and evaluate disease automatically based on several quality criteria using deep learning algorithms.

In this paper, a dataset is introduced to facilitate these advances. We call this dataset the money plant disease classification dataset. The original dataset and the augmented dataset are the two additional subfolders that make up this dataset folder. The augmented dataset was created by applying methods for data augmentation to the original data set. The Money Plant Disease Classification Dataset takes 148 MB in the folder.

In this study, mobile phone images of money plant leaves that were self-capturing were used. In the beginning, 1872 images were gathered, divided into three categories: 700 images of healthy leaf, 576 images of bacteria wilt disease leaf, and 596 images of manganese toxicity leaf. After the augmentation, we produced a total of 6000 images, 2000 of which were of healthy leaves, 2000 of which were of bacteria wilt disease, and 2000 of which were of manganese toxicity. The data were presented in RGB format in JPG format.

## 4 Experimental Design, Materials and Methods

A. Camera Specification:

A Galaxy S22 smartphone and a Redmi Note 11 Pro Plus smartphone were used to capture the data. With a sensor size of 1/1.52 inches, the 108MP Samsung ISOCELL HM2 camera on the redmi note 11 pro plus is very huge. The sensor's individual pixels are all 0.7 μm in size, but 9-in-1 binning enables the combining of 9 pixels to create a larger 2.1 μm pixel.The 50MP Samsung GN5 and the Sony IMX766 sensors on the Galaxy S22 smartphone both have a fairly large 1/1.57-inch size for the sensor. This sensor features an f/1.8 aperture and 1.0 μm single pixels.

B. Data Preprocessing:

The ability of the model to learn and grow from the input data is directly impacted by data preprocessing. Data normalization, image resizing, and noise reduction were used in the dataset's preprocessing.

**Data labelling:** We carefully labeled the data as part of the first pre-processing stage of the data, accurately categorizing each image into its appropriate category or class. Training and improvement of deep learning models depend on labelled data without accurate labels, models cannot learn and generate consistent predictions.

**Resizing:** Images have been resized to 224 × 224 pixels from 424 × 424 pixels. This is done to ensure that all images have the same size and that the network can learn similar characteristics from each one.

**Noise Reduction:** Gaussian smoothing is used to denoise the images. To avoid interfering with CNN training, this method is employed to remove any background noise from the images.

**Image segmentation**: We conducted image cropping as necessary to remove unwanted background elements, enhancing the overall quality of the dataset.

**Fig. 1:**
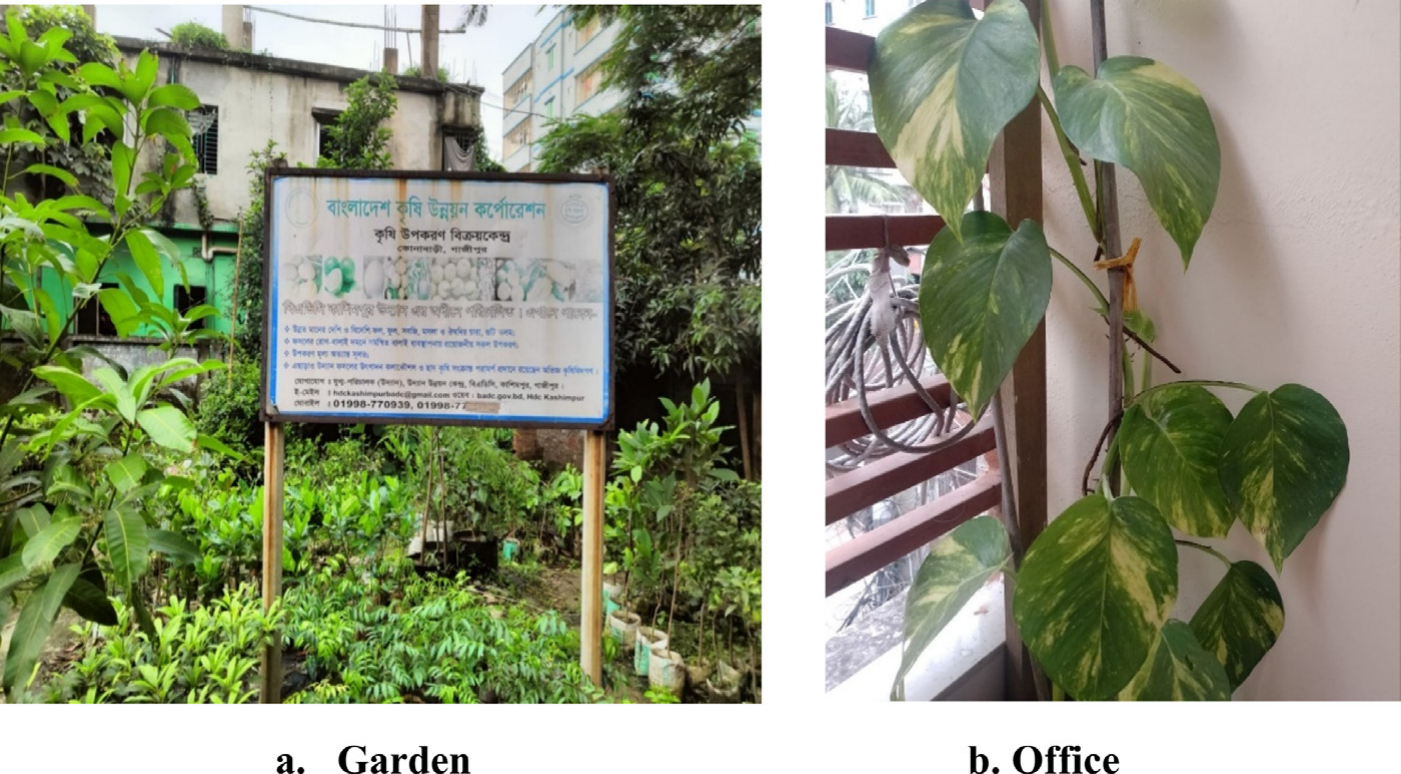
The real money plant garden, office and home from where we collected most of the dataset images.

**Fig. 2:**
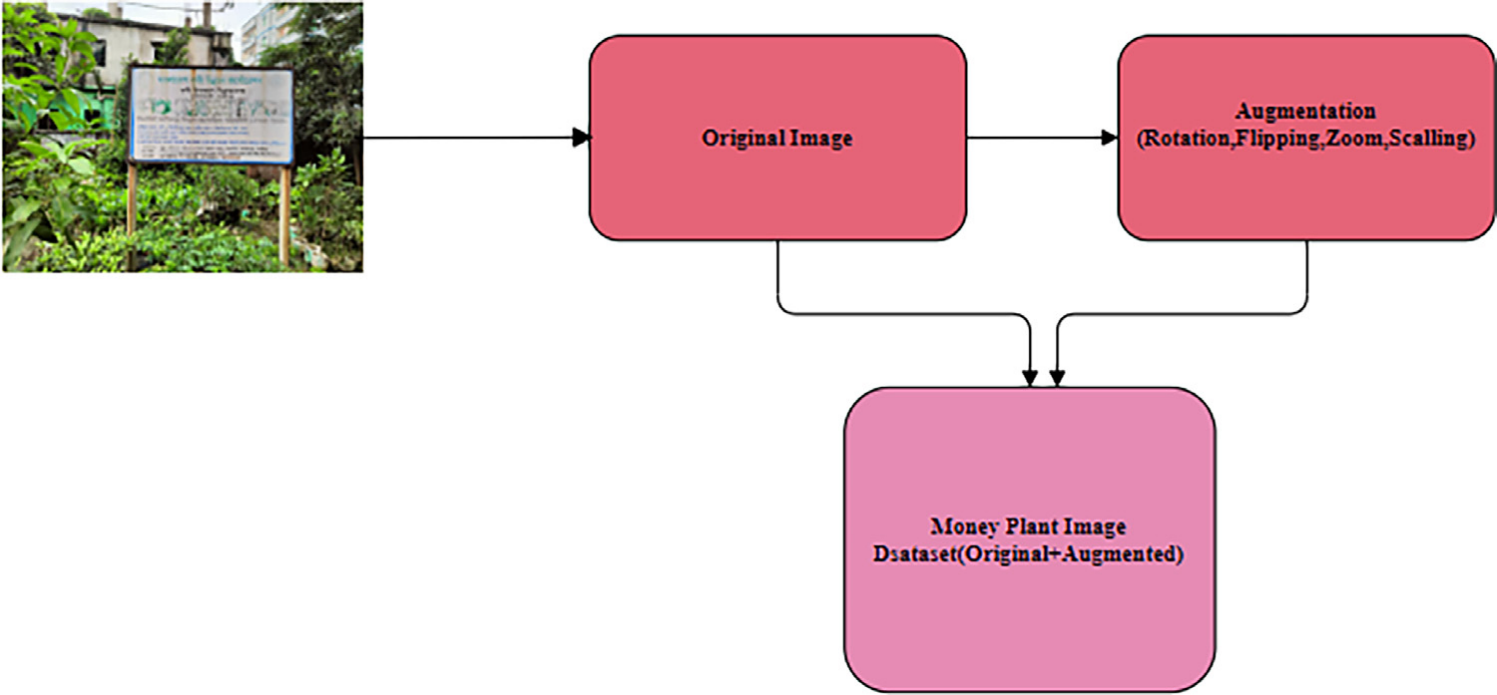
Organization of money plant disease dataset.

**Fig. 3:**
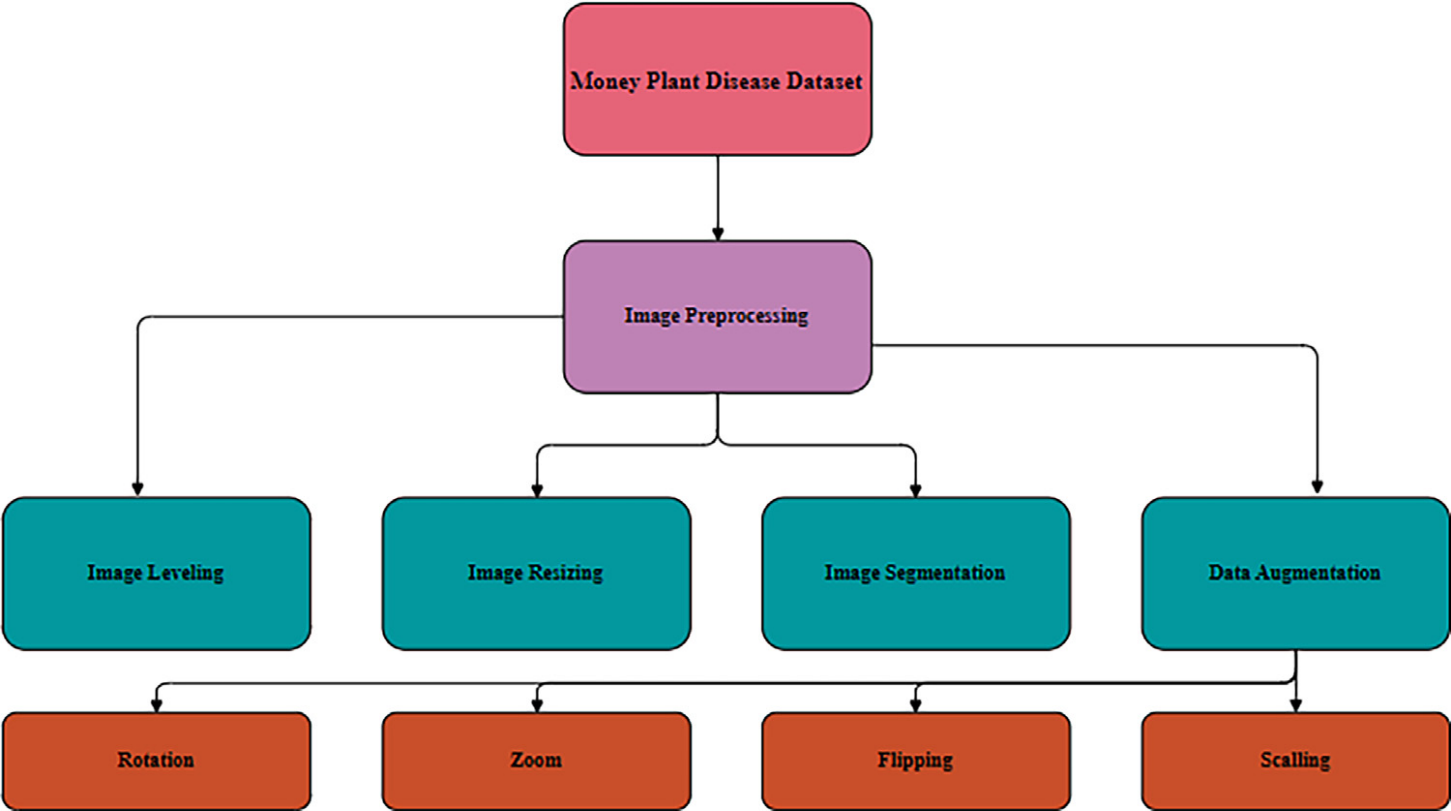
The proposed deep learning model's pre-processing stages.

**Fig. 4:**
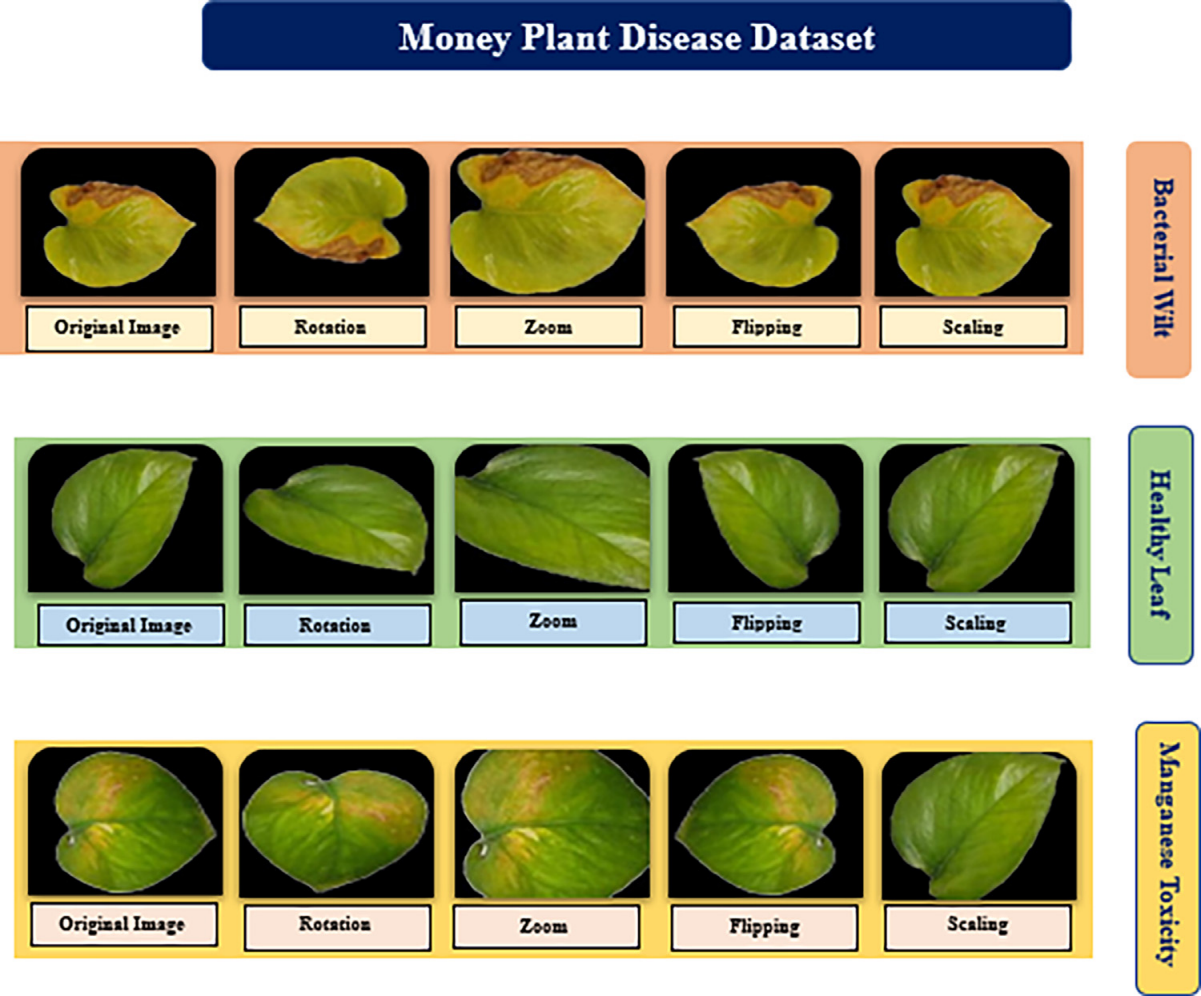
Data augmentation.

**Fig. 5:**
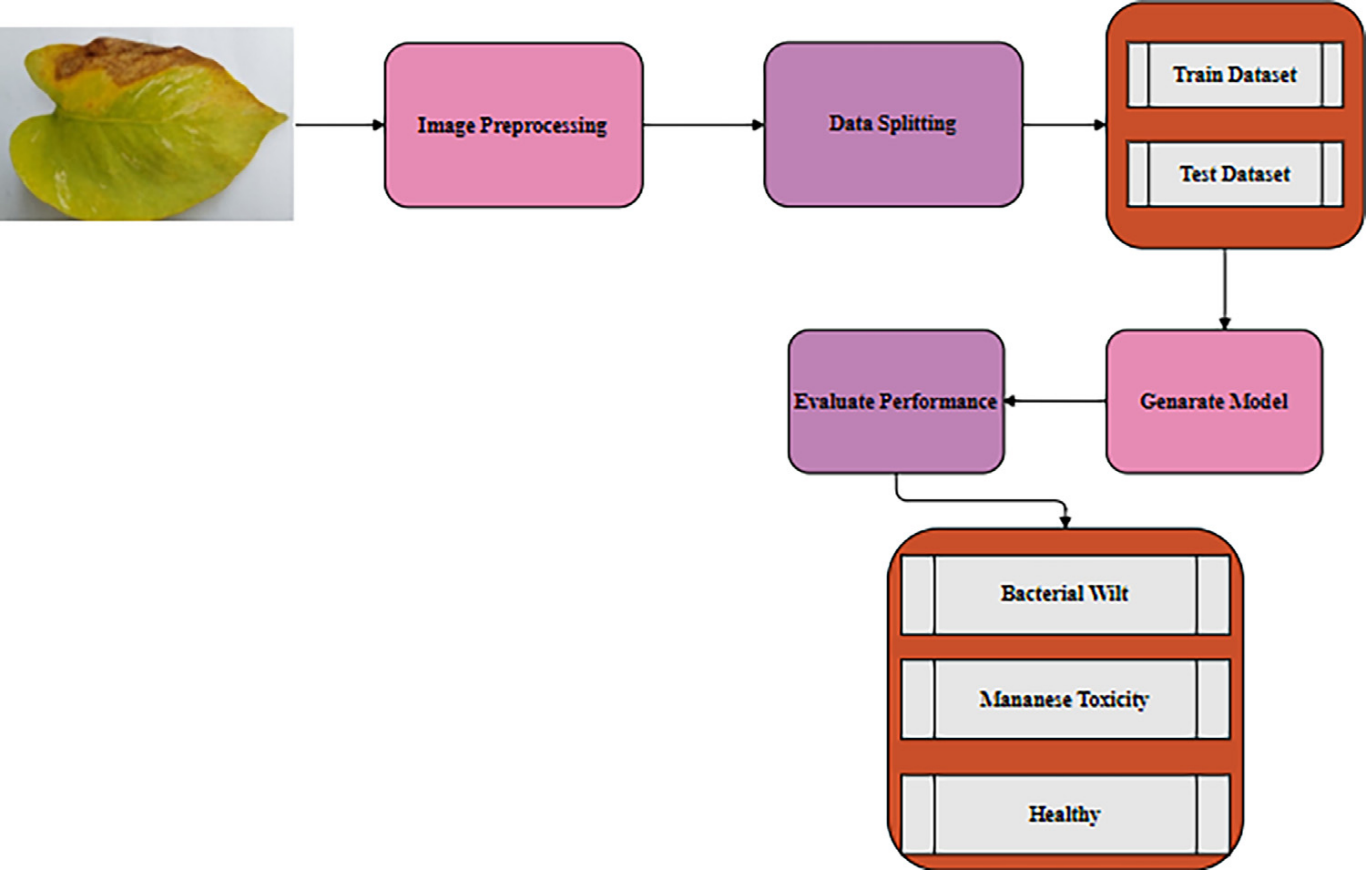
The working process for assessing money plant disease classification.

**Data Augmentation:** During our collection of money plant leaf images, we were unable to collect an equal amount of data in each class. which leads to a imbalanced dataset. Since our model is based on deep learning, a large number of images were needed. Few images in a class could affect the accuracy of the classification process. Great numbers of data are required during the deep learning training period in order to improve our experiments results. To enhance the quantity of training images, we therefore employed the image augmentation technique. Zoom (0.6, 1.1, 1.5, and min/max factors), flip left/right (0.6), and flip top/bottom (0.6) are the probability values. Rotate (max left rotation = 10, max right rotation = 20, probability = 0.6) [8]. Uniform scaling has been applied with a scaling factor ranging from 0.8 to 1.2, ensuring that both the width and height of the image are resized equally while maintaining the aspect ratio. The enhanced images include:

**Deep learning model validation:** The validation of the deep- learning model requires a comprehensive assessment of its results on a dataset. Each node in a deep learning model represents a computational unit and forms linked layers of nodes. Nodes in the input layer receive data, while nodes in the output layer produce the finished result. The hidden layers that house the neural network's primary processing power are tucked away between the input & output layers [9]. Deep learning models have achieved significant improvements in visual data analysis, particularly in areas such as object detection, natural language processing, and video and image classification [10]. The five steps of the structured five-stage process used by the model for deep learning are pre-processing of data, data segmentation, training of the model, performance assessment on a validation set & testing of the model on a separate test set. To verify the model's ability to adjust to new data and consistently produce accurate results, a precise approach is required. That's why we have used FNet in this research, which leverages Fourier transformations in place of self-attention mechanisms, making it computationally efficient for classifying this image dataset. It applies Fourier transforms to extract global features from image data, improving performance while reducing complexity, (Table 2, Table 3, Table 4, Fig. 1, Fig. 2, Fig. 3, Fig. 4, Fig. 5).

**Table 2:** Details of the dataset.

Name of class	No. of original data	No. of augmented data
Bacterial wilt	576	2000
Manganese Toxicity	596	2000
Healthy	700	2000
**Total**	**1872**	**6000**

**Table 3:** A feature of the original dataset.

Title	Description
Total Images	1872
Image Dimension	224 × 224
Color Gradings	RGB
Data Formats	JPG

**Table 4:** Evaluating our model performance with and without augmented dataset.

Model	Dataset	Precision	Recall	F1-score	Test Accuracy
FNet	Raw	0.95	0.96	0.95	95.10
	Augmented	0.99	0.99	0.99	99.95

**Fig. 6:**
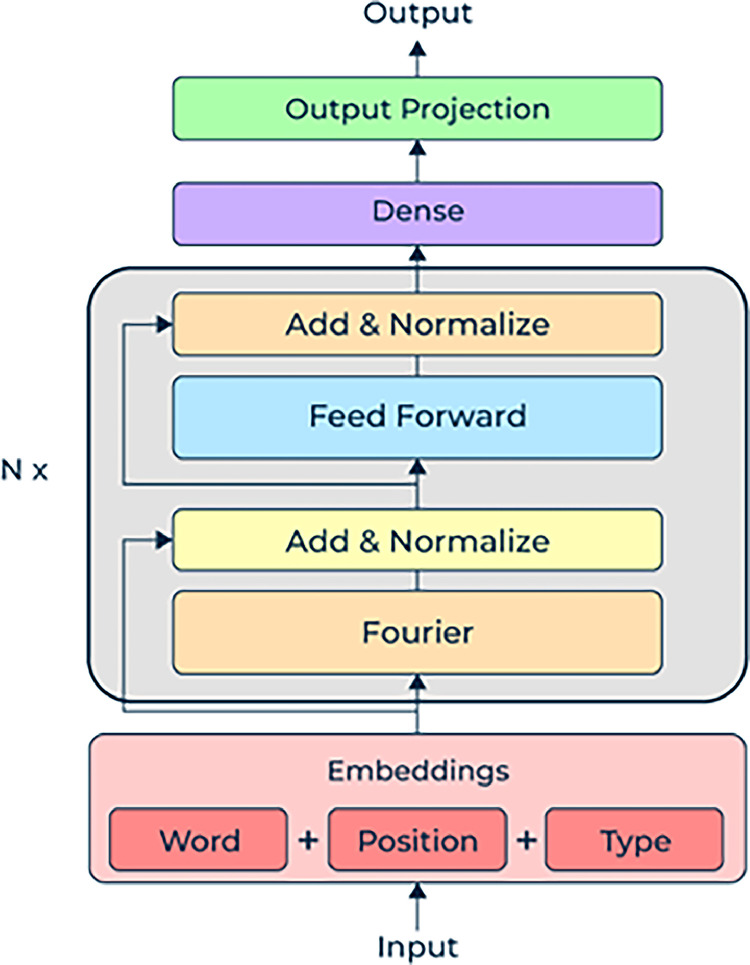
FNet architecture.

**C. Model Description:**Fig. 6 shows a neural network model architecture suited for image categorization problems. It begins with input embeddings for various image segments that incorporate pixel values, spatial information, and maybe type embeddings with varying degrees. By means of a Fourier transform, these embeddings translate spatial domain information into the frequency domain, therefore capturing intricate patterns within the image. Then by adding the transformed embeddings to the original inputs and normalising the output, a “Add & Normalise” layer stabilises the changed embeddings [11,12].

The normalised embeddings are then fed forward through a feed-forward neural network layer to learn complex interactions between several image sections. Maintaining the original input information and integrating the learnt features, another “Add & Normalise” layer improves the output from the feed-forward network. Further processing these features via a dense layer lowers dimensionality if needed and gets them ready for the output at last. After that, the output projection layer translates the processed features like the probability distribution over several image classes to the ultimate classification space. Repeated N times, the centre block which consists of the Fourier, normalising, and feed-forward layers allows the model to acquire hierarchical characteristics. Accurate image categorization is produced by this architecture effectively processing and transforming image embeddings across several levels [13,14].

**D. Measurement metrics:** In this paper, we have used the measurement metrics (accuracy, precision, recall, and F1-score) to find out how our dataset performs on the FNet model. Evaluating the success of the categorization model depends critically on these metrics. Each statistic is broken out here in a brief manner:

**Accuracy:** The accuracy of a classification model is a good measure of its generalizability. Calculated are cases exactly expected as a proportion of all the dataset occurrences. While accuracy is a good metric, especially in unbalanced datasets, it could not present a comprehensive image of model performance [[15], [16], [17], [18]].

(1) $$\text{Accuracy}=\;\frac{\text{TP}+\text{TN}}{\text{TP}+\text{TN}+\;\text{FP}+\text{FN}}$$

**Precision**: The degree to which the optimistic predictions of the model come true is measured by its precision. Out of all the expected positives, it determines the true positives percentage [[15], [16], [17], [18]].

(2) $$\text{Precision}=\;\frac{\text{TP}}{\text{TP}+\;\text{FP}}$$

**Recall:** A measure of the model's ability to find all relevant occurrences in the dataset is called recall. It is also referred to as true positive rate and sensitivity. It calculates the true positive to actual positive ratio [[15], [16], [17], [18]].

(3) $$\text{Recall}=\;\frac{\text{TP}}{\text{TP}+\;\text{FN}}$$

**F1-Score**: The F1-Score is derived from recalling and precision in which in this case false negatives and false positives are balanced. It is especially important when the false positive and false negative risks are taken into account as it takes care of multiple criteria at the same time [[15], [16], [17], [18]].

(4) $$F1\;\text{Score}=\;2\;\times \;\frac{\text{precision}\;\times \;\text{recall}}{\text{precision}+\;\text{recall}}$$

### 4.1 Confusion matrix

**Fig. 7:**
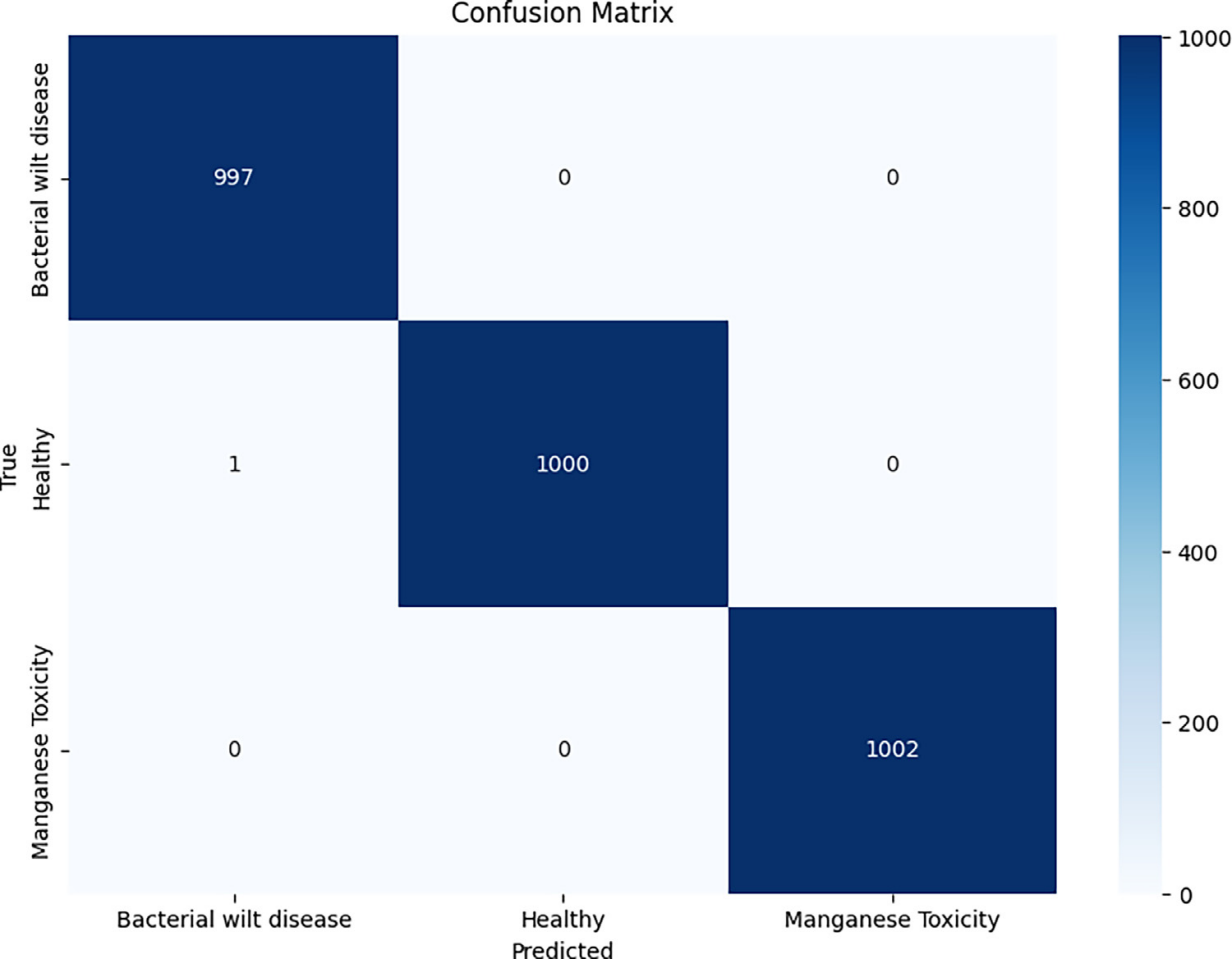
Confusion matrix of F-Net model.

The confusion matrix is an important tool for assessing the performance of classification models, particularly if multiple classes are present. This matrix helps in comprehending the degree of agreement between the predicted class and the actual or true class labels of different classes. This matrix is crucial for evaluating the model robustness of our dataset, especially by finding out the rate of misclassification of the FNet model. The suggested confusion matrix for prediction includes true positive, true negative, false positive and false negative categories and has cases/scenarios when data scientists make meaningful analyses, prioritise class performance and define causes of defects [19]. Under Fig. 7 is set the confusion matrix of Fnet model used to classify the money plant diseases's dataset. Money plant disease classification has the similar trend as Fnet successfully achieves this task. Clearly shown in Table 5, the model has performed well, classifying diseases with an impressive 99.95 % accuracy rate. This remarkable result emphasizes the model's strong generalizability, demonstrating its suitability for applications involving new data. Using this dataset, we will carefully examine advanced deep learning models in the next years to find the most efficient method for practical use [20].

**Table 5:** Classification report of money plant disease classification.

class name	Precision	Recall	F-1 score
Bacterial wilt	1.00	1.00	1.00
Manganese Toxicity	0.99	0.99	0.99
Healthy	1.00	1.00	1.00

In the future, we plan to create a consumer-focused smartphone application that will assist in the identification of money plant diseases like Bacterial wilt and Manganese Toxicity through the use of AI and machine learning techniques for image processing.

E. Analyzing performance using the best model

## Limitations

This study faces several limitations, including the reliance on smartphones for data collection, which potentially compromises image quality and uniformity. The dataset is limited to only two specific diseases and healthy Money Plant leaves, which narrows the scope of the research. Additionally, data was gathered during a single season, which may introduce temporal biases and limit the broader applicability of the findings.

## Future Work

In future we would like to enhance this work by a practical implementation through real-world testing and deployment in various environmental conditions. We also plan to expand the dataset and model capabilities to detect a broader range of diseases and apply the approach across multiple plant species. These advancements would improve the model's generalizability and practical relevance, supporting broader applications in plant health monitoring and disease management

## Ethics Statement

None of the writers of this piece have looked at any studies involving animals or people. Although everyone can access the databases used for this study, proper citation rules are still crucial.

## CRediT Author Statement

**Md. Seyam ali Biswas:** Visualization, Conceptualization, Data curation, Methodology, Validation, Writing; **MD Hasan Ahmad:** Visualization, Methodology, Data curation; **Sajib Bormon:** Methodology, Validation, Writing; **Rimon:** Writing, Data curation, review, editing; **Sohanur Rahman Sohag:** Writing, Data curation; **Amatul Bushra Akhi:** Writing –review & Supervision.

## Data Availability

Mendeley DataAdvanced Dataset on Money Plant Diseases for AI Pathology Research (Original data).
